# Layered Double Hydroxide (MgFeAl-LDH)-Based Polypropylene (PP) Nanocomposite: Mechanical Properties and Thermal Degradation

**DOI:** 10.3390/polym13193452

**Published:** 2021-10-08

**Authors:** Sajid Naseem, Sven Wießner, Ines Kühnert, Andreas Leuteritz

**Affiliations:** 1 Leibniz-Institut für Polymerforschung Dresden e.V., Hohe Straße 6, 01069 Dresden, Germany; wiessner@ipfdd.de (S.W.); kuehnert@ipfdd.de (I.K.); leuteritz@ipfdd.de (A.L.); 2 Institute of Materials Science, Technische Universität Dresden, 01062 Dresden, Germany

**Keywords:** polypropylene (PP) nanocomposite, ternary metal layered double hydroxide (LDH), catalytic degradation, MgAl and MgFeAl-LDH, transition metal LDH, thermal and mechanical properties of PP nanocomposite, rheology of LDH/PP nanocomposite

## Abstract

This work analyzes the thermal degradation and mechanical properties of iron (Fe)-containing MgAl layered double hydroxide (LDH)-based polypropylene (PP) nanocomposite. Ternary metal (MgFeAl) LDHs were prepared using the urea hydrolysis method, and Fe was used in two different concentrations (5 and 10 mol%). Nanocomposites containing MgFeAl-LDH and PP were prepared using the melt mixing method by a small-scale compounder. Three different loadings of LDHs were used in PP (2.5, 5, and 7.5 wt%). Rheological properties were determined by rheometer, and flammability was studied using the limiting oxygen index (LOI) and UL94 (V and HB). Color parameters (L*, a*, b*) and opacity of PP nanocomposites were measured with a spectrophotometer. Mechanical properties were analyzed with a universal testing machine (UTM) and Charpy impact test. The thermal behavior of MgFeAl-LDH/PP nanocomposites was studied using differential scanning calorimeter (DSC) and thermogravimetric analysis (TGA). The morphology of LDH/PP nanocomposites was analyzed with a scanning electron microscope (SEM). A decrease in melt viscosity and increase in burning rate were observed in the case of iron (Fe)-based PP nanocomposites. A decrease in mechanical properties interpreted as increased catalytic degradation was also observed in iron (Fe)-containing PP nanocomposites. Such types of LDH/PP nanocomposites can be useful where faster degradation or faster recycling of polymer nanocomposites is required because of environmental issues.

## 1. Introduction

Layered double hydroxides (LDHs), also known as hydrotalcite-like materials, are anionic clays with the formula [M_1−x_^2+^Mx^3+^(OH)_2_]^x+^·[(A^n−^)_x/n_·yH_2_O]^x^, where M^2+^, M^3+^ and A^n−^ are divalent metal cations, trivalent metal cations and interlayer anions, respectively [1,2,3]. The structure and its properties were first described by Allmann (1968) [4] and Taylor (1969) [5]. LDHs are very useful inorganic materials that have been used for many years because of their possible changes in structure, synthesis methods, and ease of preparation [3]. There are different change possibilities in their structure, such as changes in composition, type of metallic cation, interlayer anions, and combinations of different metals in LDHs [6,7]. The ease of synthesis, low costs, and natural sources attract the researcher to work on these materials [8]. There exist many techniques to prepare LDHs with a variety of metal combinations that can be custom-made to the desired applications. These custom-made LDHs have different structural, chemical, and physical properties dependent on the incorporated metals and preparation methods [9,10,11,12,13,14,15]. There are many preparation methods available for the synthesis of LDHs of which the most common are co-precipitation, urea hydrolysis, ion exchange and hydrothermal synthesis [2,16].

LDHs have been used as acid scavengers [17], in the pharmaceutical industry [18], as antioxidants [19], in biomedical applications [20,21,22,23], as UV-Vis absorption materials [12,24,25,26], in photovoltaic and solar cells [26], in supercapacitors [27], in sensors [28], in wastewater treatments [29], as precursors for photocatalytic materials [30,31], as catalysts [18,32], as stabilizers in polymers [33,34] and as flame retardants for polymers [35,36,37,38]. LDHs can be used for different purposes—as coolants and in producing diluents for flammable gas [39], as flame retardants providing resistance to ignition [40], or they can provide an alternate way of combustion with a slower rate of flame spread and can reduce the quantity of smoke [18,40]. Mostly, combinations of two metals have been used previously, and MgAl is one of the most studied combinations of LDHs used in different polymers [41,42,43,44,45].

The demand for polymeric materials has steadily increased in recent decades, and because of this increase in demand environmental pollution has also increased. With improving lifestyle, focus on the sustainable development of such polymer products is increasing. New rules and regulations on the environmental aspects of polymer products and their recyclability have encouraged researchers and manufacturers to work on environmentally friendly polymer composites or composites that can degrade faster or are easier to recycle, even in a chemical process. Biopolymers are a way to address these issues but they have the drawback of poor thermal and mechanical properties as compared to conventional polymers [46]. The higher cost of biopolymers and their difficult processing hinder their practical use in industrial and large-scale applications. Another option to address these problems is to add fillers to conventional polymers that can accelerate the degradation of polymers. These filler-based polymer nanocomposites are alternatives for sustainable development because of their low cost, easy processability, and industrial viability. Transition metal complexes can be useful for accelerated degradation of polymers such as ferric stearate, which is an effective photodegradant [47]. One such filler is layered double hydroxide (LDH) because of its multifunctional nature and its tunable structure. Adding such types of transition metal-based LDHs to polymers, which degrade the product faster after its end-use, can be useful for polymer nanocomposites. Bheki Magagula et al. used MnAl-LDH and CoAl-LDH stearate as photodegradants for LDPE and discovered that 0.1% of these active additives are sufficient to cause mechanical embrittlement of LDPE films [48].

Layered double hydroxides containing transition metals in combinations of three are the least studied in polymer nanocomposites. The use of these ternary metal LDHs in different polymers is an ongoing field of research and requires further study by preparing their polymer nanocomposites. There are very few studies available on combinations of polymers with such ternary metal LDHs, and organic modified LDHs prepared using the co-precipitation method have mostly been used in polymers. Wang et al. (2015) prepared MgAl and MgZnAl-LDH using the co-precipitation method and modified these LDHs with SDBS. They observed slightly decreased tensile strength in LDH/PP nanocomposites as compared to pure PP [49]. There are other examples in which ternary metal LDHs were used in polymers to study their effect, such as that of Nagendra et al. (2017), which studied the effect of ZnAl, CoAl, and CoZnAl-LDH on the properties of polypropylene (PP). They also used the co-precipitation method to synthesize these LDHs [50]. Gomez et al. (2019) used modified MgZnAl in polyethylene (PE), and they indicated that ternary metal (MgZnAl) can be a potential filler to protect polyethylene (PE) from UV radiations [51]. Furthermore, incorporation of a third metal ion into the layer structure can alter the LDH optical properties, UV-Vis light absorption range, and catalytic properties. Preparation methods of LDH, such as co-precipitation or urea hydrolysis, change the structure and properties of LDHs [52,53]. The incorporation of transition metals such as iron (Fe), cobalt (Co), Nickle (Ni), Copper (Cu), or zinc (Zn) in MgAl-LDH can also alter the properties of LDH, as the layer structure changes because of these transition metals. The structure of LDHs also depends on the method of preparation, whether it is co-precipitation or urea hydrolysis [6,7].

Therefore, the incorporation of LDHs prepared with different methods has different effects on polymer nanocomposites. In this research work, MgFeAl-LDHs were prepared using the urea hydrolysis method with different substitution levels of iron (Fe). The use of MgFeAl-LDH prepared via the urea hydrolysis method in polypropylene (PP) has not been discussed previously. In most of the previous research on LDH/PP composites, researchers used binary metal LDHs that were either unmodified or organically modified [54,55,56,57,58]. Thus, the focus of this work is to investigate the effect of ternary metal (MgFeAl) LDH on PP nanocomposites and to analyze their thermal degradation and mechanical properties. Ternary metal LDHs containing magnesium (Mg), aluminum (Al), and iron (Fe) were prepared with two different values of iron (Fe) substitution (5 and 10 mol%). These three types of LDH (MgFeAl(0), MgFeAl(5), and MgFeAl(10)) were used in polypropylene (PP) at the loadings of 2.5, 5, and 7.5 wt% to prepare PP nanocomposites via melt mixing method using a small-scale compounder. Different properties, including mechanical and thermal degradation, were studied for these MgFeAl-LDH/polypropylene (PP) nanocomposites to analyze whether faster degradation occurs in iron (Fe)-containing PP nanocomposites.

## 2. Materials and Methods

Chemically pure (CP) or analytical grade (AR) reactants were used for all experiments without further treatment. Mg(NO_3_)_2_·6H_2_O, Al(NO_3_)_3_·9H_2_O, and Fe(NO_3_)_3_·9H_2_O were purchased from ABCR. Urea was purchased from Sigma Aldrich. Distilled water was used for all experiments. Polypropylene (PP)(HD120MO) was purchased from Borealis A/S Denmark. Polypropylene functionalized with maleic anhydride (SCONA TPPP 2112 FA), was provided by BYK Additives & Instruments, Germany.

### 2.1. Synthesis of MgAl and MgFeAl Layered Double Hydroxides (LDHs)

MgAl and iron (Fe)-based MgFeAl-LDH were prepared by simple urea hydrolysis method as explained in the literature [6,16]. Iron (Fe) was substituted as follows: M/(Al+M) = 0.05, 0.1 for Fe (molar basis). Fe(NO_3_)_3_·9H_2_O, Mg(NO_3_)_2_·6H_2_O, and Al(NO_3_)_2_·9H_2_O salts were mixed in distilled water at the required amounts, a round bottom flask was used for solution and heated to 100 °C, the temperature was kept for 48 h. After finishing the reaction, the slurry was cooled down to room temperature and then filtered and washed with distilled water. The filtered material was dried in an oven at 70 °C for 24 h. The LDHs are designated by the following naming convention: MgFeAl(x) where x = 0 (0% Fe molar substitution), 5 (5% Fe molar substitution) and 10 (10% Fe molar substitution).

### 2.2. Preparation of MgAl and MgFeAl-LDH/PP Nanocomposites

All the nanocomposites were prepared in a small-scale compounder (Thermo Scientific-Germany, Process 11) with three different ratios of MgFeAl(0), MgFeAl(5), and MgFeAl(10)-LDH as shown in Table 1. The compatibilizer/PP ratio was kept constant in the different formulations. The temperature used was 190 °C and the screw rotation speed was set to 100 rpm. Specimens for tensile strength, impact strength, and fire testing were generated by injection molding using a Dr. Boy 22 A HV (Dr. Boy Machine Incorporation, Germany). The sample names and their LDH/PP amounts are shown in Table 1. The pictures of prepared samples are also shown in Table 1.

### 2.3. Characterization Methods

Color parameters (L*, a*, b*) (DIN 6174) and opacity (ISO 2471) were measured using the spectrophotometer CM-3600A (Konica Minolta). The limiting oxygen index (LOI) measurements were carried out using an oxygen index meter (FTT, UK) following ASTM D2863-19. The UL94 V and UL94 HB testing was carried out according to the method provided previously to determine the burning rate of the PP nanocomposites [42]. Rheological characteristics of PP nanocomposites were studied in oscillatory shear using a strain-controlled rheometer (ARES, Rheometrics Scientific, USA). The experiments were performed over a frequency range of 0.1 to 100 rad/s at 180 °C and 5% strain. Tensile tests were done with a Zwick 1456 (model 1456, Z010, Ulm Germany) (length: 82 mm, width: 10 mm, thickness: 2 mm) with a crosshead speed of 50 mm min^−1^ according to DIN EN ISO 527-2/1BA/50, including the modulus testing at a crosshead speed of 1 mm/min up to 0.25% elongation. The Charpy impact strength was measured by PSW 15J using the standard ISO 179/1eU. The results were averaged over five measurements for each sample for the flammability investigation, tensile testing, and Charpy impact testing. All the specimens prepared for flammability and mechanical testing were injection molded under identical conditions at 180 °C. Scanning electron microscopy (SEM) images of samples were taken with a Zeiss Ultra Plus. The samples for SEM analysis were cut with an Ultramicrotom UC7 from Leica at −180 °C. The thermal characterization of LDH/PP nanocomposites was determined using thermogravimetric analysis (TGA) and differential scanning calorimetry (DSC). TGA measurement was performed at a heating rate of 10 °C/min using a TGA Q5000 from TA instruments in an inert nitrogen atmosphere and air atmosphere. The temperature range of 25 to 800 °C was used for TGA analysis. Differential scanning calorimeter (DSC) analysis was done using DSC Q2000 from TA instruments. Three cycles of heating-cooling-heating with a heating/cooling rate of 10 °C/min and sample size of about 5 mg were used. The thermograms of the second heating cycle were analyzed for the study of the melting behavior of the samples.

## 3. Results and Discussion

### 3.1. Color Parameters (L*, a*, b*) and Opacity Measurement

The color of pure PP was almost transparent, and the color of MgFeAl(0)/PP nanocomposites was turbid, which increased as the amount of MgFeAl(0)-LDH increased. The colors of MgFeAl(5)/PP and MgFeAl(10)/PP nanocomposites were light brown to dark brown from a low amount to a high amount of LDH. The amount of iron (Fe) also caused a darker color as it increased from 5 to 10 mol%, as can be seen in Table 1. The values of opacity and color parameters (L*, a*, b*) measured by spectrophotometer are shown in Table 2, which indicated that samples of polypropylene (PP) showed some scattering because of the semi-crystalline nature of PP. The color impression on the red–green axis was as close to unity as can be expected, whereas the value on the blue-yellow axis already indicated a small shift to yellow, which can also be expected of once processed PP. With the addition of filler all color values changed. As the amount of MgFeAl(0)-LDH increased from 2.5 to 7.5 wt% in PP composites, the values of opacity increased because of the increase in scattering phenomena related to the dispersed LDH particles. Furthermore, the iron (Fe)-based LDH caused an even darker color in PP nanocomposites from the presence of the transition metal and the related absorption of light. The value of opacity increased even more as the amount of MgFeAl(5)-LDH and MgFeAl(10)-LDH increased, compared to the sample containing no iron, as can be seen in Table 2.

### 3.2. Limiting Oxygen Index (LOI) Test, UL94 Test, and Burning Behavior of Different LDH/PP Nanocomposites

The limiting oxygen index (LOI) provides basic information about fire retardancy for any polymeric nanocomposites [59]. The influence of different LDH systems and different concentrations of loading on values of the LOI for LDH/PP nanocomposites is shown in Table 3. Pure PP has the LOI value of 20 and with the addition of different LDHs the LOI increases. LOI values increased from 20 (pure PP) to 21.0, 22.4 and 21.9 in cases of MgFeAl(0)/PP, MgFeAl(5)/PP, and MgFeAl(10)/PP, respectively. The increase in the LOI in all the systems of LDH/PP was only slight, as the level of loadings was not as large as previously studied LDH/polymer systems [42]. During the LOI test, the flame propagated vertically downward, and flame propagation took place through the surface to the core of the LDH/PP nanocomposites. Pure PP burned like a candle with continuous dripping of melt until the whole sample burned, while the LDH/PP nanocomposites showed different behavior, as three regions were identified on the burning surface. The skin layer of nanocomposites consisted of char and then melt layer supported by a solid layer. The convective flow of mass in the melt layer occurred with the bubbling of gaseous material because of the decomposition of LDHs. Because the loading levels of the LDHs were not high enough to make the thicker char layer in these nanocomposites, the difference was not large. Francis Costa et al. (2007) used a higher concentration of MgAl-LDHs, which reached 20 wt% in polyethylene, and the LOI increased from 18 to 22. In the case of 2.5 and 5 wt%, there was no increase in the LOI, and in the case of 7.5 wt%, a 0.7 increase in the LOI was observed [42]. Therefore, the inclusion of transition metal-based MgFeAl-LDH provided a greater increase in the LOI in PP nanocomposites at 7.5 wt%, although the effect was small.

#### UL94 Test

The UL94 test followed two standards (UL94 V and UL94 HB). None of these LDH/PP nanocomposites passed UL94 V test specifications (vertical burning test standard). This was expected as the amount of LDH used in PP was very low in this research, and there was neither an organic modification nor a synergistic flame retardant present in the composition. Compared with previous investigations of MgAl-LDHs in polyethylene (PE) up to 16.2 wt%, a similar result was observed [42]. Previously studied Clay/PP nanocomposites also showed similar results, and there were no UL94 ratings achieved in that case, either [60]. All the prepared MgAl and MgFeAl-LDH/PP nanocomposites started burning after 10 s and burned completely up to the clamp holder. As the addition of LDHs changed the melt viscosity, especially at higher loading, the dripping behavior also changed. Iron (Fe)-containing MgAl-LDH decreased the melt viscosity of PP nanocomposites, and these results can be interlinked with the rheological analysis shown in Section 3.3. In the rheological analysis as shown in Figure 2 the viscosity decreased in Fe-containing MgAl-LDH-based PP nanocomposites.

**Figure 1:**
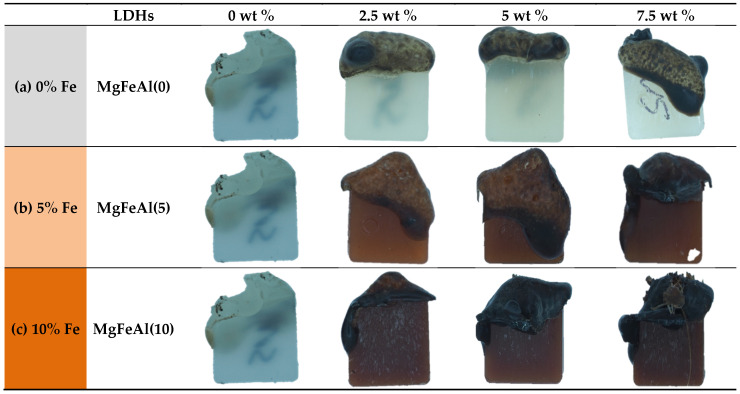
Burning behavior of different loading level of LDHs/PP nanocomposites with different iron (Fe) substitutions: (**a**) 0, (**b**) 5, and (**c**) 10%.

UL94 HB (horizontal burning test standard) revealed that iron (Fe)-substituted MgAl-LDH/PP nanocomposites showed a faster burning rate as compared to pure PP and MgAl/PP nanocomposites. The melt viscosity was low in the case of MgFeAl/PP nanocomposites as compared to MgAl/PP nanocomposites. The burning rate values are shown in Table 4. The burning rate increased because the viscosity of the LDH/PP nanocomposites was not high enough to hold the burned material/char, and so it dripped away from the sample, creating a new surface, and the new material started burning easily [42]. The Fe present in the nanocomposites decreased the melt viscosity, while burning those samples showed a higher burning rate. Figure 1a–c shows images of samples after the burning test, and it can be observed that the color of char became darker as the amount of Fe increased in LDH.

### 3.3. Rheological Analysis of Different LDH/PP Nanocomposites

**Figure 2:**
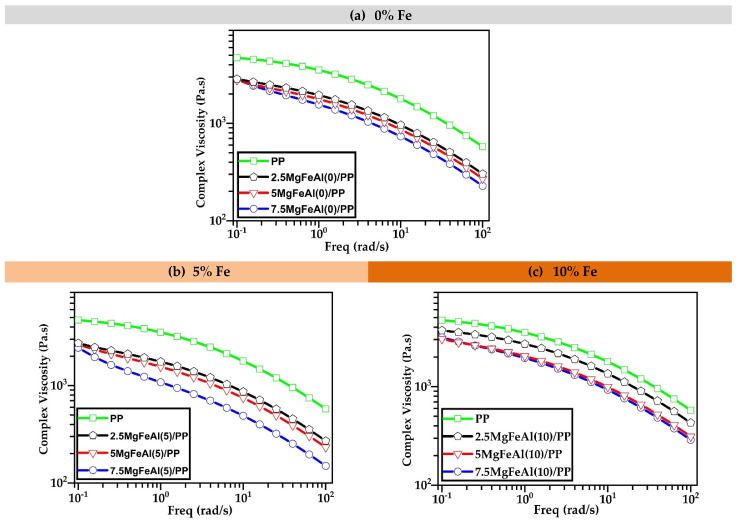
Rheological analysis of different LDH/PP nanocomposites performed at 180 °C: (**a**) 0, (**b**) 5, and (**c**) 10% Fe.

To understand the particle dispersion of LDH in PP nanocomposites and provide information about its melt processability, it was important to analyze the rheological behavior of these tri-metal LDH-based PP nanocomposite systems [61]. Melt rheological properties of LDH/PP nanocomposites depend on the interaction of tri-metal LDH particles with PP. The complex viscosities of pure PP and MgFeAl(0)/PP, MgFeAl(5)/PP, and MgFeAl(10)/PP nanocomposites are shown in Figure 2a–c. From Figure 2a–c it can be observed that the addition of LDH decreased the viscosity of the LDH/PP nanocomposites. The decrease in viscosity was more prominent in MgFeAl-LDH as compared to MgAl-LDH. As the amount of MgAl and Fe-based MgFeAl-LDH increased, the viscosity decreased further. This was related to the increased mobility or enhanced relaxation of PP chains [41,61].

The complex viscosity decreased more in the case of MgFeAl(5)-based PP nanocomposites from 2.5 to 7.5 wt% as compared to MgFeAl(0)/PP nanocomposites. The reason for the decrease in complex viscosity was an increase in free volume and dilution effect caused by iron (Fe)-based MgFeAl-LDH. The other reason was that degradation reactions in the PP occurred because of melt processing and the addition of the compatibilizer. As the amount of iron (Fe) increased, the amount of degradation in processing grew, since compatibilizer and processing at high temperatures enhance the expected catalytic effect of iron (Fe). Previously, Wang et al. studied polypropylene (PP)/Mg_3_Al–tartrazine-layered double hydroxide (LDH) nanocomposites and observed a similar behavior of decrease in viscosity of the composites [61]. They found that when the average particle separation distance is smaller than twice the polymer radius of gyration *Rg*, then the nanoparticles will disturb polymer chain configurations and lead to a decrease in the viscosity [61].

### 3.4. Mechanical Properties (Tensile Testing and Impact Testing)

The mechanical properties of LDH/PP nanocomposites are shown in Table 5. Tensile strength decreased with the addition of MgFeAl(0), MgFeAl(5), and MgFeAl(10)-LDHs as compared to pure PP. On the other hand, the modulus increased steadily as the amount of LDHs increased. The effect of compatibilizer was also relevant for LDH/PP nanocomposites when studying the tensile properties [62]. The reinforcing nature of MgAl-LDH or MgFeAl-LDH was more prominent when LDH modified with organic anions was used in higher amounts in PP. Impact strength also decreased as the amount of MgFeAl(0), MgFeAl(5), and MgFeAl(10)-LDH increased. As the amount of MgFeAl(0) increased from 2.5 to 7.5 wt% the tensile strength increased from 33.7 to 35.1 MPa. On the other hand, in the cases of MgFeAl(5) and MgFeAl(10), the tensile strength decreased steadily as the amount increased from 2.5 to 7.5 wt%. Impact strength also decreased with the addition of LDH, as can be seen in Table 5. The impact strength was 110 kJ/m^2^ for pure PP and 47.5, 35.9, and 44.4 kJ/m^2^ for 7.5 MgFeAl (0), 7.5 MgFeAl (5), and 7.5 MgFeAl (10), respectively. The decrease in mechanical properties was greater in the case of MgFeAl (5)/PP nanocomposites because of enhanced PP-degradation due to Fe during processing, and this was supported by the decrease in viscosity in rheological analysis. The substitution of Fe in MgAl-LDH changed the structure of the layers of LDH, as studied and discussed previously [6,7]. This change in structure by using transition metal substitution (Fe) changed the charge on LDH layers and caused the catalytic degradation when mixing with PP at higher temperature, eventually causing the decrease in tensile strength and impact strength.

### 3.5. Thermogravimetric Analysis (TGA) of MgFeAl(0), MgFeAl(5). and MgFeAl(10) PP Nanocomposites

**Figure 3:**
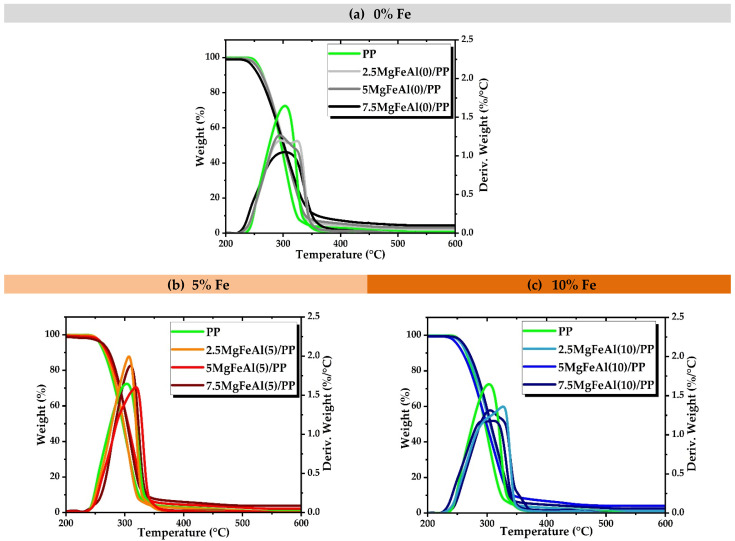
TGA analysis of different LDH/PP nanocomposites in nitrogen environment showing the effect of different amounts of Fe: (**a**) 0, (**b**) 5, and (**c**) 10%.

The influence of the different metal ion combinations of LDHs and various loadings of different LDHs on the thermal behavior of PP nanocomposites was determined with TGA analysis. TGA analysis results of the decomposition in nitrogen for PP nanocomposites containing 0, 5, and 10% Fe of different loadings are shown in Figure 3a–c. The addition of Fe into the MgAl-LDH structure resulted in a difference in the thermal behavior of nanocomposites, as can be observed in Figure 3a–c.

**Figure 4:**
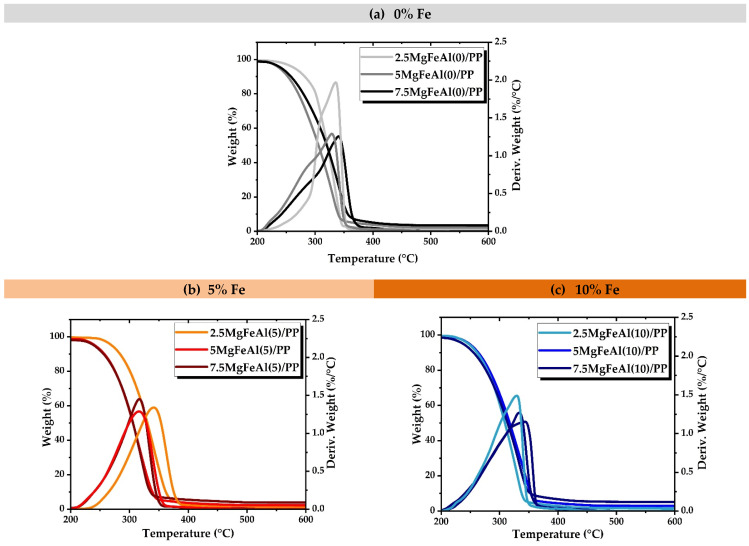
TGA analysis of different LDH/PP nanocomposites in air environment showing the effect of different amounts of Fe: (**a**) 0, (**b**) 5, and (**c**) 10%.

The TGA curves for the LDH/PP nanocomposites are very similar to that of the pure PP with the difference in decomposition rate. The TGA study of Fe-containing LDHs revealed a more complex behavior as compared to MgAl-LDH. As seen in the TGA, traces of a catalytic effect on degradation can be expected in Fe-containing MgFeAl-LDH/PP nanocomposites [63]. Figure 4a–c shows the TGA results of nanocomposites studied in an air environment. As the substitution level of Fe increased from 5 to 10% in MgAl-LDHs, a difference in weight loss rate in PP nanocomposites was also observed. The peaks shifted toward lower temperature in the case of Fe-containing LDH/PP nanocomposites, as can be seen in Figure 4b. As the substitution level of iron (Fe) increased in the LDH, the thermal stability of PP nanocomposites decreased [60,64]. As the substitution level of Fe increased in MgAl-LDHs the surface area was also enhanced, and the platelet sizes of the LDH particles decreased because the Fe_2_O_3_ phase increased as the substitution level increased [52]. The difference in loading level also affected the thermal behavior, and results are shown in the Supplementary files (Figures S1 and S2). The residue after TGA analysis contained not only carbonaceous materials but also some metal oxides, which are observed in the dark color of char in Figure 1a–c [42].

### 3.6. Differential Scanning Calorimetry (DSC) Analysis of Different LDH/PP Nanocomposites

**Figure 5:**
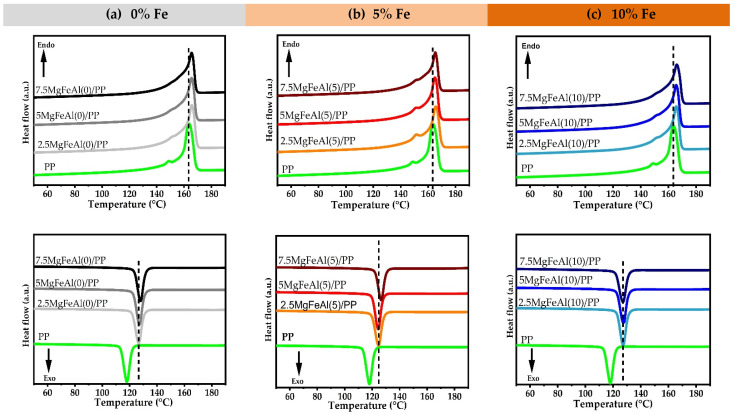
DSC thermograms of different LDH/PP nanocomposites: (**a**) 0, (**b**) 5 and (**c**) 10% Fe.

Figure 5a–c shows the DSC thermograms of different LDH/PP nanocomposites. The addition of LDH into PP resulted in higher crystallization peak temperature, which indicated a higher nucleation activity. The structure of MgFeAl-LDH was different from that of MgAl-LDH and the substitution of Fe^3+^ increased the *d(003)* value and “c ” parameter; the detailed structural analysis was explained in a previous article [6]. The small increase in LDH layer spacing and the change in structure could be the reasons for higher nucleation and the higher crystallization of Fe-containing LDH/PP nanocomposites, assuming a better match of structural parameters in PP and LDH [62]. The small increase in interlayer spaces could have increased the crystallization temperature (T_c_), as shown previously in the cases of LDH and MMT [64]. The change in crystallization behavior was more prominent with the grreater increase in the interlayer spacing and organomodification, as compared to the change in only the layer structure, which was the case in this research. The replacement of Al^+3^ with Fe^+3^ in the MgAl-LDH structure changed the charge structure of LDH layers more, as compared to the interlayer spaces [62,65].

### 3.7. Scanning Electron Microscopy (SEM) Images and Morphological Analysis of Different LDH/PP Nanocomposites

**Figure 6:**
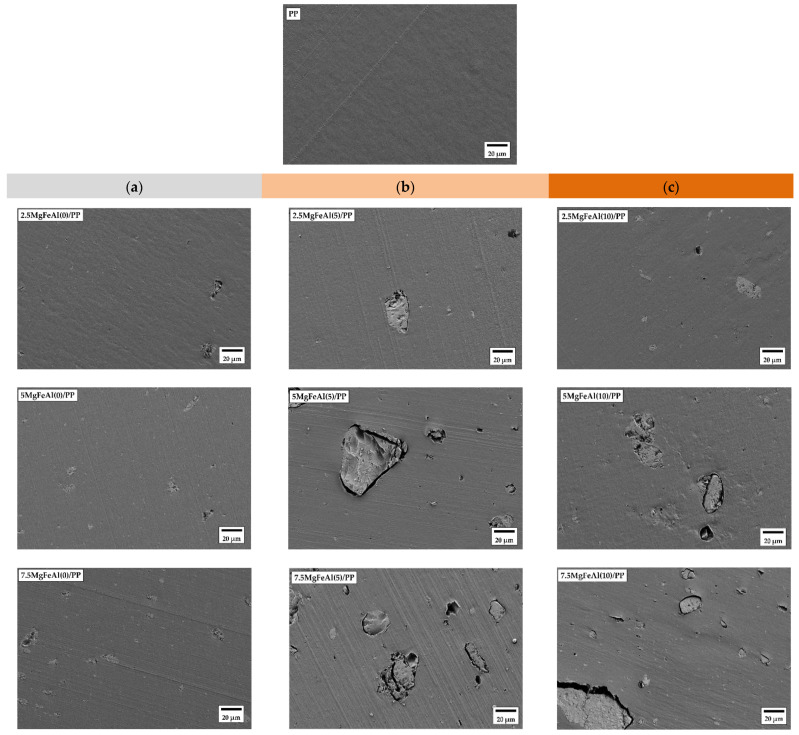
SEM images of pure polypropylene (PP), and (**a**) MgFeAl(0)/PP (2.5, 5, 7.5 wt%), (**b**) MgFeAl(5)/PP (2.5, 5, 7.5 wt%), (**c**) MgFeAl(10)/PP (2.5, 5, 7.5 wt%) nanocomposites.

Figure 6a–c shows the SEM images of pure polypropylene (PP) and different LDH/PP nanocomposites with 2.5, 5, and 7.5 wt% loadings. Figure 6a–c shows the SEM images of MgFeAl(0), MgFeAl(5), and MgFeAl(10), respectively. Particles of LDH can be seen in all the LDH/PP nanocomposites. As the amount of LDH increased, the agglomeration of LDH particles increased, which led to poor dispersion and a decrease in the mechanical properties of nanocomposites [66,67]. As the amounts of MgFeAl(5) and MgFeAl(10) increased, the agglomeration of LDH particles increased and led to even lower dispersion in PP. This led to lower mechanical strength. The amount of ternary metal LDH as well as the substitution percent of iron (Fe) could also change the thermal degradation of MgFeAl/PP nanocomposites. The Fe-based ternary metal MgFeAl-LDH needed organic modification to enhance the miscibility of MgFeAl and PP. In such an effort, the metal activity then cannot be traced back to the metal alone, but also to an interaction with the anion used for modification. This can lead to different thermal degradations of PP in modified MgFeAl-LDH as compared to unmodified MgFeAl-LDH. The iron (Fe)-based LDH can then cause degradation to PP but with poor miscibility, as seen in the SEM images.

## 4. Conclusions

The effect of MgFeAl-LDH on the thermal degradation and mechanical properties of polypropylene (PP) nanocomposites was analyzed. MgFeAl-LDH was synthesized using the urea hydrolysis method, and nanocomposites of PP were prepared using the melt mixing method. The effect and interaction of Fe-containing MgAl-LDH and Fe-free MgAl-LDH in PP nanocomposites were analyzed and compared in this research work. MgAl-LDH substituted with two different amounts of Fe, 5 and 10%, was compounded with polypropylene (PP) at filling levels of 2.5, 5, and 7.5%. It is known that iron (Fe) substitution in MgAl-LDH can change the layered structure, particle size, and surface area of LDH. Here, because of the presence of Fe, the thermal behavior changed and showed a lower degradation temperature than Fe-free LDH polypropylene (PP) nanocomposites, indicating a catalytic degradation. Additionally, because of the change in layer structure, a higher effect on nucleation was determined in the DSC investigation. Rheological measurement led to lower viscosity, which was interpreted as degradation combined with relaxation phenomena. The flammability study and rheological study emphasized the catalyzing effect of Fe, which enhanced the burning rate of MgFeAl/PP nanocomposites and decreased the melt viscosity. The integration of LDH resulted in the decrease in tensile strength, which was more pronounced in the case of Fe-containing composites, emphasizing a catalytic effect on degradation during processing. Such types of MgFeAl-LDH/PP nanocomposites can be useful where faster degradation and recycling of polymer products are required, such as in the packaging industry. From the SEM micrographs, it was also observed that mixing was poor because of the agglomeration of MgFeAl-LDH, and organic modification can be useful if these types of ternary metal LDHs must be used in higher amounts in PP or other polymers.

## Figures and Tables

**Table 1:** Sample details of LDH/PP nanocomposites with their pictures.

Sr. No.	LDHs	Amount (wt%)	PP (wt%)	Sample Color
1	-	0	100	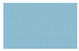
2	**MgFeAl(0)**	2.5	97.5	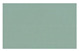
3		5	95	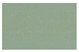
4		7.5	92.5	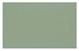
5	**MgFeAl(5)**	2.5	97.5	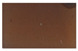
6		5	95	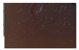
7		7.5	92.5	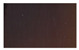
8	**MgFeAl(10)**	2.5	97.5	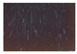
9		5	95	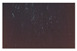
10		7.5	92.5	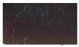

**Table 2:** Color parameters (L*, a*, b*) and opacity measured by spectrophotometer.

Sr. No.	LDHs	Amount (wt%)	Color Parameters (L*, a*, b*)	Opacity
1	-	0	65.3, 0.81, 6.75	22.53
2	**MgFeAl(0)**	2.5	55.08, 1.62, 14.08	41.98
3		5	51.81, 2.14, 13.64	48.83
4		7.5	48.59, 2.33, 12.69	60.64
5	**MgFeAl(5)**	2.5	32. 38, 11.16, 9.6	88.82
6		5	33.16, 8.08, 7.18	96.72
7		7.5	35.55, 9.33, 7.55	96.06
8	**MgFeAl(10)**	2.5	30.82, 9.36, 7.64	97.59
9		5	30.67, 8.86, 7.31	98.88
10		7.5	35.92, 7.71, 6.33	101.25

**Table 3:** Limiting oxygen index (LOI) of different LDH/PP nanocomposites.

LDHs Contents (%)	Limiting Oxygen Index (LOI)		
	MgFeAl(0)/PP	MgFeAl(5)/PP	MgFeAl(10)/PP
0	20 ± 0.08	20 ± 0.08	20 ± 0.08
2.5	21.6 ± 0.15	21.7 ± 0.07	21.4 ± 0.11
5	20.8 ± 0.1	21.8 ± 0.1	21.7 ± 0.11
7.5	21 ± 0.08	22.4 ± 0.11	21.9 ± 0.15

**Table 4:** Burning rate of different LDH/PP nanocomposites.

LDH Contents (%)	Burning Rate (mm/min)		
	MgFeAl(0)/PP	MgFeAl(5)/PP	MgFeAl(10)/PP
0	14.2 ± 0.17	14.2 ± 0.17	14.2 ± 0.17
2.5	16.3 ± 0.03	19.8 ± 0.13	15.7 ± 0.28
5	14.9 ± 0.15	17.1 ± 0.29	13.6 ± 0.35
7.5	14.2 ± 0.03	15.3 ± 0.09	12.8 ± 0.49

**Table 5:** Mechanical properties of different LDH/PP nanocomposites.

Sr. No.	LDH/PP Nanocomposites	Youngs Modulus (MPa)	Tensile Strength (MPa)	Charpy Impact Strength (kJ/m^2^)
1	PP	1340 ± 32	37.3 ± 1.5	110 ± 11
2	2.5 MgFeAl(0)	1630 ± 60	33.7 ± 0.4	57.6 ± 5.4
3	5 MgFeAl(0)	1710 ± 100	34.3 ± 0.8	55.4 ± 4.3
4	7.5 MgFeAl(0)	1850 ± 100	35.1 ± 1.1	47.5 ± 4.5
5	2.5 MgFeAl(5)	1630 ± 120	35.1 ± 0.5	57.0 ± 3.3
6	5 MgFeAl(5)	1670 ± 80	34.6 ± 0.4	47.9 ± 4.8
7	7.5 MgFeAl(5)	1800 ± 80	34.5 ± 0.6	35.9 ± 7.6
8	2.5 MgFeAl(10)	1650 ± 60	34.1 ± 0.6	69.8 ± 3.9
9	5 MgFeAl(10)	1670 ± 40	33.5 ± 0.6	55.3 ± 3.1
10	7.5 MgFeAl(10)	1700 ± 140	33.5 ± 0.3	44.4 ± 3.4

## Data Availability

Not applicable.

## Supplementary Materials

The following are available online at https://www.mdpi.com/article/10.3390/polym13193452/s1, Figure S1. TGA analysis of different LDH/PP nanocomposites in nitrogen environment showing the effect of amount of Fe (a) 2.5% LDH (b) 5% LDH (c) 7.5% LDH; Figure S2. TGA analysis of different LDH/PP nanocomposites in air environment showing the effect of amount of Fe (a) 2.5% LDH (b) 5% LDH (c) 7.5% LDH.
