# Supplementary material for: Layered Double Hydroxide (MgFeAl-LDH)-Based Polypropylene (PP) Nanocomposite: Mechanical Properties and Thermal Degradation
Source: Polymers (Basel). 2021 Oct 8;13(19):3452. doi: 10.3390/polym13193452 (PMC8512664; doi:10.3390/polym13193452)
Supplement: Supplementary file 1 [file polymers-13-03452-s001.zip › polymers-1411731-supplementary.pdf]

Supplementary material

Layered Double Hydroxide (MgFeAl-LDH)-Based Polypropylene (PP) Nanocomposite: Mechanical Properties and Thermal Degradation

Sajid Naseem [1,2] *, Sven Wießner [1,2], Ines Kühnert [1], Andreas Leuteritz [1]

[1] Leibniz-Institut für Polymerforschung Dresden e.V., Hohe Straße 6, 01069 Dresden, Germany; naseem@ipfdd.de (S.N.); wiessner@ipfdd.de (S.W.); kuehnert@ipfdd.de (I.K.); leuteritz@ipfdd.de (A.L.)

[2] Institute of Materials Science, Technische Universität Dresden, 01062 Dresden, Germany

* Correspondence: naseem@ipfdd.de; Tel.: +49 351 4658 689

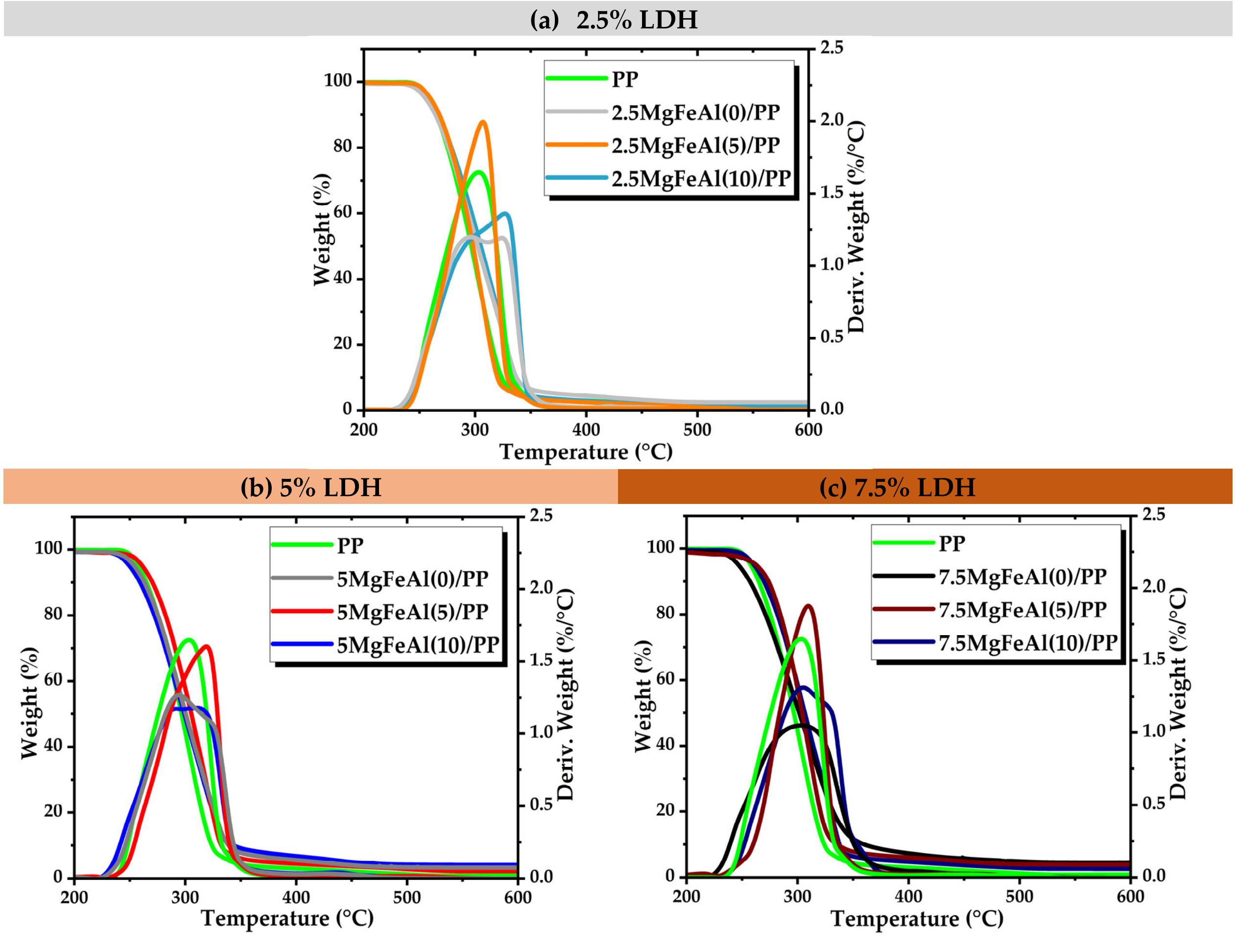

Figure S1. TGA analysis of different LDH/PP nanocomposites in nitrogen environment showing the effect of amount of Fe (a) 2.5 % LDH (b) 5% LDH (c) 7.5% LDH

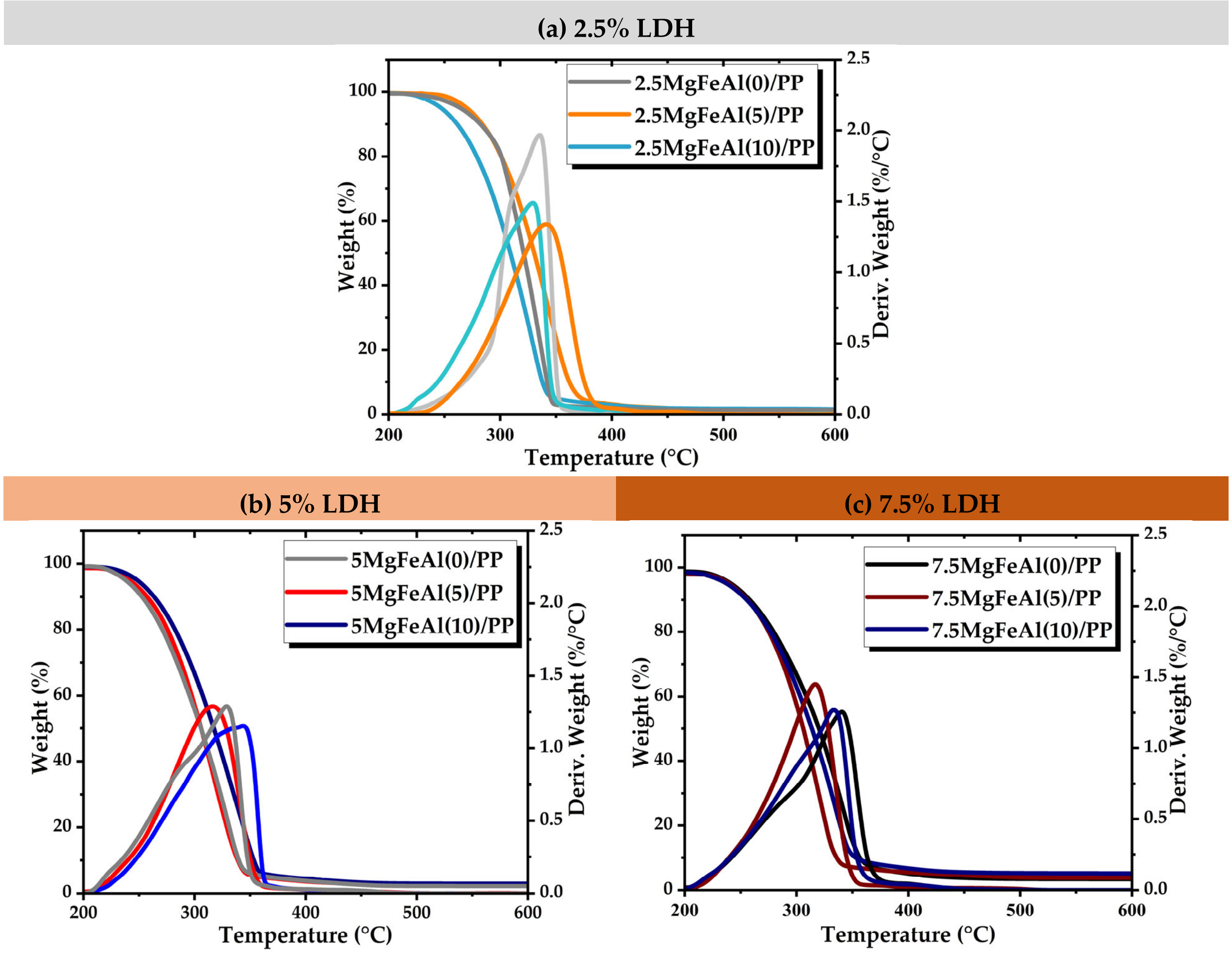

Figure S2. TGA analysis of different LDH/PP nanocomposites in air environment showing the effect of amount of Fe (a) 2.5 % LDH (b) 5% LDH (c) 7.5% LDH.
